# Cannabis-associated hyperalgesia and reduced local anesthetic efficacy in oral surgery: A case report

**DOI:** 10.1016/j.toxrep.2026.102300

**Published:** 2026-06-27

**Authors:** Susan Hammoud, Mirian Harumi Itikawa Magno, Joseph Akhikar, Luiz Carlos Magno Filho

**Affiliations:** a The University of Detroit Mercy School of Dentistry, Detroit, MI, United States; b Private practice – Glenview, IL, United States

**Keywords:** Cannabis use, Hyperalgesia, Anesthetic resistance, Oral surgery, Tetrahydrocannabinol, Pain modulation, Local anesthetic efficacy

## Abstract

Cannabis use has increased significantly in recent years, yet its impact on local anesthetic efficacy in oral surgery remains poorly understood. This report analyzes the case of a 34-year-old male with history of chronic cannabis use who exhibited a hyperalgesic pain response and difficulty achieving adequate anesthesia during dental extraction in spite of standard anesthetic techniques. This atypical presentation may be explained by cannabis-associated alterations in pain pathways, including hyperalgesia related to chronic cannabinoid exposure, pharmacokinetic, and hemodynamic effects. This case highlights the potential impact of cannabis on pain perception and anesthetic response, underscoring the need for clinicians to consider cannabis use when planning anesthesia and to adapt management strategies accordingly.

## Highlights

• This case report documents cannabis-associated hyperalgesia and diminished local anesthetic efficacy during oral surgery extraction in a chronic user, despite conventional techniques.
• Chronic THC exposure likely induces CB1 receptor downregulation and TRPV1 modulation, precipitating heightened nociception, tachycardia, and CYP450-mediated pharmacokinetic alterations affecting lidocaine metabolism.
• Preoperative cannabis use screening, alternative agents like buffered articaine, and maximum dosing vigilance are essential, warranting prospective studies amid escalating use prevalence.

## Graphical Abstract

**Figure:**
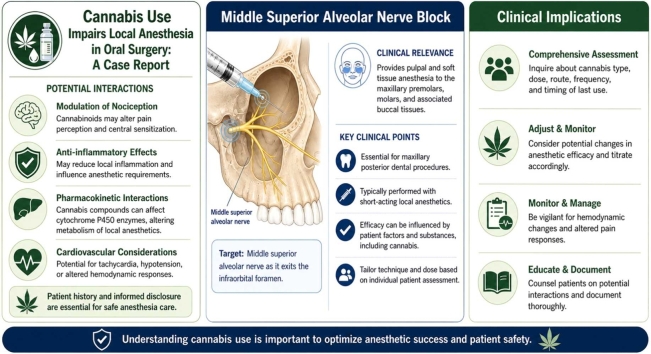

## 1 Introduction

Cannabis use across the United States has risen significantly in recent years. Resultantly, providers are increasingly encountering an individual with chronic cannabis use in the perioperative setting. While cannabis has been utilized for analgesic and anxiolytic effects, its impact on anesthetic management remains poorly understood and is often unpredictable [1].

Cannabis contains hundreds of biologically active constituents, among which Δ⁹-tetrahydrocannabinol (THC) and cannabidiol (CBD) are the two most prominent cannabinoids. THC is the principal psychoactive component of cannabis and is largely responsible for the euphoric and intoxicating effects associated with its use. In contrast, CBD is a major non-psychoactive constituent that has been shown to exert anticonvulsant, analgesic, and anti-inflammatory effects [2].

Pharmacologically, cannabinoids bind to receptors known as cannabinoid receptors (CB1 and CB2), which are predominantly expressed in the cardiovascular, neurological, gastrointestinal, immunological, and respiratory systems. The observed pharmacological effects are largely attributable to the action of THC, which may increase the risk of tachycardia, cardiac arrhythmia, bronchodilation, nausea, vomiting, impaired fertility, alterations in thyroid hormone levels, cognitive disturbances, psychotic episodes, behavioral changes and hyperalgesia [3].

Hyperalgesia is characterized by heightened pain perception in response to a normally painful stimulus. Emerging evidence suggests that cannabis use may alter pain perception, anesthetic requirements, and physiologic responses during procedures. Chronic cannabis use has been associated with episodes of hyperalgesia, complicating perioperative pain management. These cases frequently necessitate escalated opioid administration as rescue therapy to achieve adequate analgesia [2].

This case describes a hyperalgesic response and difficulty achieving adequate local anesthesia in a patient with recent cannabis use undergoing tooth extraction.

## 2 Case report

34-year-old African American male presented to the Oral and Maxillofacial Surgery Department at the University of Detroit Mercy with a chief complaint of pain on the left side of the face. Clinical examination revealed tooth #13 with extensive coronal caries. Periapical radiographic evaluation demonstrated deep carious involvement extending into the root and pulp, consistent with a diagnosis of acute irreversible pulpitis associated with acute apical periodontitis.

The patient’s medical history was significant for attention-deficit/hyperactivity disorder (ADHD) and generalized anxiety disorder, both diagnosed in 2017 and not managed pharmacologically. His social history included daily electronic cigarette use for two years, conventional cigarette smoking once daily for five years, alcohol consumption twice weekly (two to three drinks per occasion), and daily recreative cannabis use since the age of 29. During the consultation, the patient presented with mild agitation, conjunctival injection, dry mouth and euphoria.

Prior to the procedure, the patient was asked about his most recent cannabis use; however, he reported being unsure of the exact timing. Preemptive medication consisted of ibuprofen 800 mg and a loading dose of amoxicillin 1 g, administered one hour prior to the procedure. The treatment plan involved extraction of tooth #13 under local anesthesia, and informed consent was obtained.

Topical anesthesia was achieved using 20% benzocaine gel applied to the mucosa adjacent to teeth #12, #13, and #14 after drying the site with gauze. The gel remained in place for five minutes. Local anesthesia was initiated using 2% lidocaine with epinephrine 1:100,000 via buccal infiltration targeting the middle superior alveolar (MSA) nerve, with an injection rate of approximately 1 mL/min. After confirming negative aspiration, anesthetic administration was initiated.

Within seconds of injection onset, the patient experienced sudden and intense pain, prompting immediate cessation of the procedure. The patient's condition rapidly progressed with a marked increase in sensitivity. He subsequently reported throbbing pain radiating across the left side of the face and exhibited unusual behavior, including compressing the affected side, inappropriate laughter, and abruptly standing from the dental chair.

After several minutes, the patient became calmer but continued to report significant discomfort. The anesthetic was then changed to 4% articaine with epinephrine 1:100,000, buffered with 0.18 mL of 8.4% sodium bicarbonate, following the protocol described by Bala et al. [4]. Following administration, the patient reported progressive pain relief and improved comfort.

Supplemental anesthesia was administered in the palatal region using the same buffered solution. Approximately five minutes later, the patient again reported sudden severe pain in the region of tooth #13, necessitating an additional cartridge of buffered articaine delivered via both buccal and palatal infiltrations. Adequate anesthesia was subsequently achieved. The surgical procedure proceeded uneventfully. Tooth #13 was extracted using luxators and forceps #150, followed by placement of a collagen membrane and suturing.

## 3 Discussion

Despite the use of standard anesthetic techniques, this case demonstrates an atypical outcome not readily explained by established causes of local anesthetic failure, known drug interactions, or underlying medical conditions. However, this presentation is consistent with emerging reports in an individual with chronic cannabis use, suggesting altered pain thresholds and anesthetic responses to local anesthetic. The growing number of these emerging cases remains largely unexplored, and the phenomenon remains poorly characterized, particularly within the field of dentistry. As cannabis use becomes more prevalent, understanding its potential impact on perioperative pain management is increasingly important for dental and surgical providers [1], [2], [5].

This case adds to the increasing body of evidence suggesting that cannabis use may alter pain pathways. Similar to hyperalgesia observed with long-term opioid use, cannabis may contribute to heightened pain sensitivity. While cannabis has historically been perceived to have analgesic properties, an individual of long term cannabis use grows, emerging research suggests that long-term or heavy use may paradoxically result in increased pain perception [1], [6]. Chronic exposure to cannabinoids, particularly THC, has been associated with adaptive changes in the endocannabinoid system, including receptor desensitization and downregulation, which may diminish endogenous pain modulation over time.

Central sensitization has been proposed as a mechanism underlying pain hypersensitivity in a variety of chronic pain conditions and is characterized by amplification of neural signaling within central nociceptive pathways, resulting in exaggerated responses to painful stimuli. Such alterations in pain processing may provide a useful framework for understanding the atypical presentation observed in this case [10].

Cannabis exerts its effects through exogenous cannabinoids, primarily THC and CBD, which interact with CB1, CB2, and transient receptor potential (TRP) channels involved in nociception. Activation of CB1 receptors influences neurotransmitter release that alter pain perception, CB2 receptors are more closely associated with inflammatory responses, and TRPV1 receptors are involved in the detection of noxious stimuli and thermal pain. 2 The clinical implications of these pathways as they relate to anesthetic efficacy, and the interactions between these systems not well understood, particularly in the context of cannabinoid exposure. Chronic exposure to THC has been preliminarily associated with downregulation and desensitization of CB1 receptors, which may contribute to altered central pain processing and paradoxical hyperalgesia [7].

Concurrently, cannabinoids have been shown to modulate TRPV1 receptor activity, a central mediator of nociceptive signaling and peripheral pain sensitivity [8]. This interaction may result in altered pain thresholds and exaggerated responses to noxious stimuli, including to local anesthetic injection as was seen in this case. Importantly, central sensitization has been shown to manifest clinically as hyperalgesia and exaggerated responses to noxious stimuli. These responses may occur despite relatively limited peripheral pathology and may result in pain presentations that appear disproportionate to the provoking stimulus [10]. While these mechanisms remain incompletely understood, they provide a biologically plausible explanation for the atypical pain response observed.

Compoundingly, acute exposure or withdrawal from substances has been associated with increased sensitivity to pain and stress, a concept that could potentially apply to cannabis [3], [7]. In this case, the patient’s uncertain timing of last use followed by later admission of recent intake raises the possibility that either acute intoxication or early withdrawal could have been contributors to the exaggerated response.

An interesting consideration in this case is the presence of irreversible pulpitis, a persistent inflammatory disease of the dental pulp characterized by irreversible structural and functional damage. Resultantly, those patients frequently require supplemental anesthetic techniques and repeat administration. Furthermore, inflammatory sensitization associated with irreversible pulpitis is known to produce clinical presentations that mimic other pain disorders through generating diffuse and referred pain responses, including TMJ, TMD, and neuropathic pain disorders. Therefore, the patient's underlying pulpal pathology likely contributed to the pain experienced and the anesthetic challenges encountered during treatment. However, while irreversible pulpitis represents an important competing explanation, the abrupt onset, severity, and behavioral nature of the patient's response appeared atypical when compared with clinical courses commonly observed. Most notably, contradictory to typical anesthetic failures of inflamed pulp in which gradual development of inadequate anesthesia is typically observed, this patient experienced immediate hyperalgesic response to the injection. Thus, further pointing towards central sensitization factors. As such, it is possible that cannabis-associated alterations in pain processing acted synergistically with the existing inflammatory condition, amplifying an already sensitized pain state and resulting in the exaggerated presentation observed in this case [10], [11].

From a pharmacokinetic perspective, interactions between local anesthetics and cannabinoid remain poorly studied [3]. Cannabinoids are known to influence cytochrome P450 enzyme activity, potentially altering drug metabolism. Both THC and CBD have been shown to inhibit multiple CYP450 isoenzymes, including CYP3A4, CYP2C9, and CYP2C19, with CBD demonstrating more potent inhibitory effects. These interactions may alter the metabolism of concurrently administered medications, potentially leading to changes in drug plasma levels and duration of action. While local anesthetics such as lidocaine are partially metabolized by CYP450 enzymes, the clinical significance of cannabinoid-mediated CYP inhibition on local anesthetic metabolism remains unclear. Altered enzymatic activity may contribute to variability in anesthetic onset, efficacy, and duration. Additionally, chronic cannabis use may induce adaptive changes in hepatic enzyme activity, further complicating the predictability of drug metabolism in these patients. Although direct evidence linking CYP450 modulation by cannabinoids to local anesthetic failure is limited, this mechanism represents a plausible contributing factor to the atypical anesthetic response observed in this case [9].

Furthermore, well established cannabis-induced vasodilation and reflex tachycardia may result in increased systemic absorption of local anesthetics, thereby reducing efficacy at the target site. THC has been shown to induce peripheral vasodilation through CB1 receptor–mediated effects on vascular smooth muscle, as well as through modulation of autonomic tone. This vasodilatory response is often accompanied by reflex tachycardia, likely secondary to decreased systemic vascular resistance, as well as compensatory sympathetic activation [1], [5]. Thus, potentially explaining the need for repeated anesthetic administration observed in this case.

We hypothesize that this patient’s cannabis use contributed to both hyperalgesia and difficulty achieving adequate anesthesia. This case underscores the need for increased clinician awareness of potential anesthetic challenges in patients who use cannabis, particularly with chronic use [1], [2], [5]. Thorough preoperative screening, including specific inquiry into the timing, frequency, and method of cannabis use, is essential. Providers should be prepared to modify anesthetic strategies, including the use of alternative agents or buffering techniques, while remaining mindful of maximum safe dosing to avoid toxicity.

## 4 Conclusion

Overall, this case contributes to the growing body of literature suggesting that cannabis use may impact anesthetic efficacy and pain response. Given the increasing prevalence of cannabis use, further research is needed to better characterize these interactions and to develop evidence-based guidelines for managing affected patients in oral and maxillofacial surgery.

## CRediT authorship contribution statement

**Joseph Akhikar:** Writing – original draft, Validation. **Mirian Harumi Itikawa Magno:** Resources, Formal analysis. **Luiz Carlos Magno Filho:** Writing – original draft, Project administration, Investigation, Conceptualization. **Susan Hammoud:** Writing – original draft, Data curation.

## Funding

None

## Disclosures

None

## Declaration of Competing Interest

The authors declare that they have no known competing financial interests or personal relationships that could have appeared to influence the work reported in this paper.

## Data Availability

No data was used for the research described in the article.
